# Long-term outcomes of aortic grafts after 2-week storage in a rat model of orthotopic aortic transplantation

**DOI:** 10.1007/s00210-026-05368-9

**Published:** 2026-04-29

**Authors:** Manuel J. Santander, Narges Waezi, Ursula Rauen, Niels Voigt, Bernhard C. Danner, Ingo Kutschka, Samuel Sosalla, Ahmad Fawad Jebran, Tomislav Stojanovic

**Affiliations:** 1 Department of Thoracic and Cardiovascular Surgery, University Medical Center Göttingen, Robert Koch Strasse 40, 37075 Göttingen, Germany; 2 DZHK (German Center for Cardiovascular Research), Partner Site Göttingen, Göttingen, Germany; 3 Department of Vascular Surgery and Endovascular Surgery, Hospital Kassel, Kassel, Germany; 4 Institute of Physiological Chemistry, University Hospital Essen, Essen, Germany; 5 Institute of Pharmacology and Toxicology, University Medical Center Göttingen, Göttingen, Germany; 6 Department of Cardiac Surgery, University Hospital Basel, Basel, Switzerland; 7 Department of Cardiology, Kerckhoff Heart Center, Bad Nauheim, Germany; 8 Department of Cardiology and Angiology, University Hospital Giessen & Cardio-Pulmonary Institute (CPI), Justus-Liebig-University Giessen, Excellence Cluster, Giessen, Germany; 9 Department of Vascular Surgery and Endovascular Surgery, Hospital Wolfsburg, Wolfsburg, Germany

**Keywords:** Cold storage, Ischemia-reperfusion, Intimal hyperplasia, Bypass grafts, Graft vasculopathy, Orthotopic aortic transplantation

## Abstract

Vascular allografts represent a potential alternative to autologous grafts when suitable vessels are unavailable. However, current preservation methods, particularly cryopreservation, lead to substantial endothelial damage and poor clinical outcomes. This study evaluated the long-term efficacy of an N-acetylhistidine-buffered, potassium-chloride-enriched and amino-acid-fortified solution augmented with iron chelators, as an advanced preservation solution (NTK-Chel), compared to histidine-tryptophan-ketoglutarate (HTK) and normal saline for vascular graft storage. Abdominal aortae from male Wistar rats (*n* = 75) were stored for 2 weeks at 4 °C in NTK-Chel (*n* = 24), HTK (*n* = 26), or normal saline (*n* = 25), then transplanted orthotopically. Grafts were harvested at 8, 16, and 26 weeks post-transplantation for vascular function testing and histological analysis. Upon explantation, smooth muscle contractility was severely impaired in all preservation solutions at 8, 16, and 26 weeks post-transplantation. At 8 weeks, only NTK-Chel-preserved grafts demonstrated detectable contractile responses to KCl, though these did not meet criteria for comprehensive vasomotor testing. By 16 and 26 weeks, all grafts had lost measurable contractility. The NTK-Chel group was associated with significantly reduced intimal hyperplasia compared to HTK and normal saline, particularly at 8 and 26 weeks. However, progressive smooth muscle cell loss occurred in all groups, with NTK-Chel showing only delayed rather than prevented degeneration. While NTK-Chel resulted in significantly less intimal hyperplasia compared to conventional solutions, prolonged cold storage combined with transplantation-associated remodeling ultimately compromises long-term vasomotor function regardless of preservation strategy.

## Introduction

Autologous vascular grafts remain the gold standard for revascularization in cardiovascular surgery due to superior long-term outcomes (Dorigo et al. 2012; Head et al. 2017; Ambler and Twine 2018). With the growing prevalence of cardiovascular disease, demand for vascular tissue grafts continues to increase (GBD 2013 Mortality and Causes of Death Collaborators 2015). However, autologous vessel tissue is often unavailable due to poor quality, inadequate size, or prior use in revascularization procedures (McPhee et al. 2012; Hartranft et al. 2014). In such cases, allografts represent a potentially valuable alternative.

Currently, cryopreservation is the most commonly used method for preserving vascular allografts, offering the advantage of “off-the-shelf” availability (O’Banion et al. 2017). However, cryopreservation causes extensive endothelial destruction, which promotes graft failure (Lehle et al. 2006). Clinical outcomes have consequently been disappointing, with poor short- and long-term patency rates (Hartranft et al. 2014; O’Banion et al. 2017). At present, cryopreserved grafts are primarily recommended for vascular reconstruction in the setting of infection when autologous grafts are unavailable (Harlander-Locke et al. 2017; Bossi et al. 2017). To prevent graft failure and improve patency, the concept of optimal graft preservation has been extensively discussed in the literature over recent decades (Schaeffer et al. 1997; Fahner et al. 2006; Winkler et al. 2016; Woodward et al. 2016). Vascular grafts are typically stored under hypothermic conditions (4 C) in either normal saline or organ preservation solutions such as Histidine-Tryptophan-Ketoglutarate (HTK). However, hypothermia exerts dual effects on vascular tissue. While it protects against cell damage by slowing enzymatic activities and reducing metabolic demand during ischemia (Guibert et al. 2011), it can simultaneously induce significant injury in certain cell types, particularly endothelial cells, by triggering iron-dependent production of highly reactive oxygen species (Rauen et al. 1999, 2000; Rauen and de Groot 2004).

To further improve vessel preservation, a modified HTK-based storage solution, referred to as NTK-Chel in this study, was developed. NTK-Chel is a potassium-enriched, N-acetylhistidine-buffered, amino acid-fortified solution containing deferoxamine and LK 614 as iron chelators (Wille et al. 2008; Wille and Rauen 2010). Several in vitro animal studies using pig and rat aorta as well as rat mesenteric artery have demonstrated superior preservation of endothelial function and endothelium-smooth muscle coupling with NTK-Chel compared to HTK and normal saline, with this advantage persisting even after 21 days of storage (Wille et al. 2008; Zatschler et al. 2009). Given that vessel banking protocols ideally require storage durations of approximately 2 weeks, this represents a potentially significant advance (Garbe et al. 2011). Additional in vitro experiments using clinically relevant human graft materials, internal mammary artery and saphenous vein, have confirmed NTK-Chel’s improved preservation capacity compared to HTK and normal saline (Garbe et al. 2011; Wilbring et al. 2011a, b).

To date, only short-term in vivo experimental studies have investigated the protective performance of NTK-Chel following brief storage periods of 2 h. In these studies, aortic grafts stored in NTK-Chel demonstrated significantly better endothelial function and integrity after 1 week compared to HTK and normal saline (Veres et al. 2016). However, to establish the clinical relevance of NTK-Chel as a preservation solution, an in vivo model incorporating prolonged storage times and long-term implantation data is essential. We therefore designed an experimental in vivo rat model of orthotopic infrarenal aortic transplantation to evaluate whether 2-week cold storage of aortic grafts in NTK-Chel improves endothelial integrity, vascular contractility, and long-term graft patency compared with HTK and normal saline.

## Materials and methods

### Animals and ethical approval

This study was approved by the German ethical committee of the Lower Saxony State Authority for Protection of Animals (reference number 33.11.42502–04/019/09). Inbred male Wistar rats weighing 300–350 g were obtained from Charles River Laboratories (Sulzfeld, Germany) and used as donors and recipients. All animals were treated in accordance with German legislation on protection of animals and housed under standard laboratory conditions with free access to standard chow and water ad libitum. Animals were maintained on a 12-h light/dark cycle at 20 ± 2 °C. All experiments were conducted in accordance with the ARRIVE (Animal Research: Reporting of In Vivo Experiments) guidelines.

### Experimental design

**Fig. 1:**
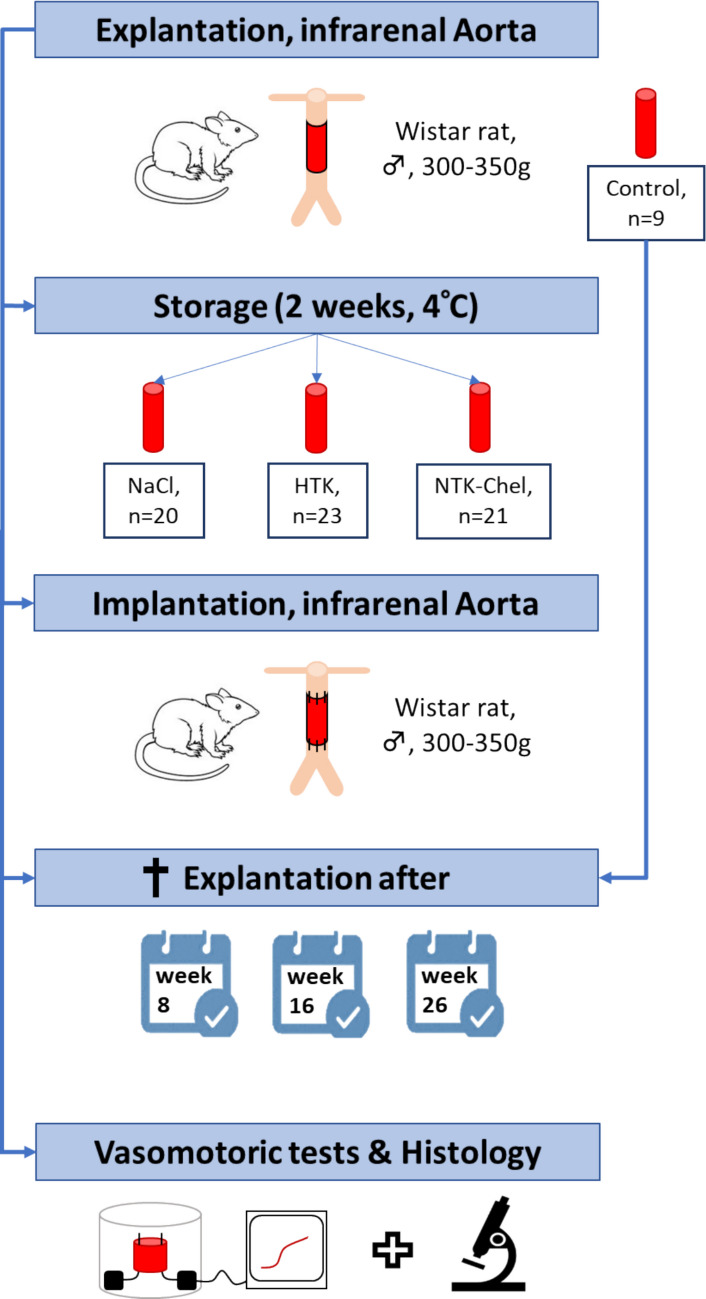
Experimental workflow of the study. The infrarenal abdominal aorta was explanted from donor rats and flushed with cold preservation solution. Grafts were stored for 2 weeks at 4 °C in NTK-Chel, HTK, or NaCl. Control grafts were analyzed immediately after explantation. After cold storage, grafts were orthotopically implanted into the infrarenal abdominal aorta of recipient rats using end-to-end anastomoses. Grafts were explanted after 8, 16, or 26 weeks and subjected to vasomotor function testing and histological analysis. Abbreviations: NaCl, normal saline; HTK, histidine-tryptophan-ketoglutarate; NTK-Chel, *N*-acetylhistidine-buffered storage solution enriched with potassium chloride, containing additional amino acids and iron chelators

Aortic grafts from Wistar rats were harvested and allocated to three preservation groups and one control group (Fig. 1). Explanted grafts were preserved for 2 weeks at 4 °C in normal saline (0.9% NaCl), histidine-tryptophan-ketoglutarate solution (HTK; Custodiol®, Dr. Franz Köhler Chemie GmbH, Bensheim, Germany), or NTK-Chel (obtained from Dr. Franz Köhler Chemie GmbH). NTK-Chel is a modified HTK-type solution supplemented with iron chelators (deferoxamine and the hydroxamic-acid derivative LK 614) and corresponds to the formulation of solution 8 in Wille et al. 2008 (Table 1). In the baseline control group, aortic segments were harvested and immediately processed for vasomotor function testing and histological analysis without storage or transplantation.

**Table 1:** Composition of the preservation solutions used in this study. Component concentrations are provided in mmol/L for histidine-tryptophan-ketoglutarate solution (HTK) and the modified HTK-type solution supplemented with iron chelators (NTK-Chel), according to the manufacturers’ specifications. The hyphen “-” indicates that the component is not present or not specified for the respective solution

Component	HTK (mmol/L)	NTK-Chel (mmol/L)
Sodium chloride	15	14
Potassium chloride	9	73
Magnesium chloride 6 H₂O	4	8
Disodium hydrogen phosphate	-	1
Calcium chloride 2 H₂O	0.015	0.05
Histidine	180	-
Histidine HCl H₂O	18	-
*N*-acetylhistidine H₂O	-	30
Tryptophan	2	2
α-Ketoglutaric acid	-	2
Potassium hydrogen 2-ketoglutarate	1	-
Mannitol	30	-
Sucrose	-	20
Glucose monohydrate	-	10
Aspartic acid	-	5
Glycine	-	10
Alanine	-	5
Potassium hydroxide	-	20
Deferoxamine mesylate	-	0.1
3,4-Dimethoxy-N-methylbenzohydroxamic acid (LK 614)	-	0.02

In each preservation group, grafts were orthotopically transplanted after 2 weeks of cold storage and subsequently harvested at 8, 16, or 26 weeks post-transplantation. A subset of aortic rings from each explanted graft was subjected to vasomotor function testing (Table 2), while all explanted grafts underwent histological and immunohistochemical analysis.

**Table 2:** Distribution of aortic grafts per experimental group and follow-up time. The table summarizes the total number of grafts per group and the subset of grafts subjected to vasomotor function testing at 8, 16, and 26 weeks after implantation. All explanted grafts were processed for histological analysis. Abbreviations: *NaCl* normal saline, *HTK* histidine-tryptophan-ketoglutarate, *NTK-Chel N*-acetylhistidine-buffered storage solution enriched with potassium chloride, containing additional amino acids and iron chelators

Groups	Total (*n*)	8 weeks (*n*)	16 weeks (*n*)	26 weeks (*n*)
NaCl	19	8	5	6
Assessment of vasomotor function	13	4	5	4
HTK	22	8	8	6
Assessment of vasomotor function	15	5	5	5
NTK-Chel	21	10	5	6
Assessment of vasomotor function	15	5	5	5
Baseline	9	3	3	3
Assessment of vasomotor function	9	3	3	3

### Surgical procedures

We employed a well-established model of orthotopic infrarenal aortic transplantation. This approach was chosen over heterotopic transplantation to minimize technique-related influences on graft patency, such as diameter mismatch or flow turbulence.

All animals were anesthetized with sevoflurane and maintained on controlled heating pads throughout the procedure. Buprenorphine (0.05 mg/kg) was administered subcutaneously prior to surgery and, in recipient animals, was continued for 3 postoperative days for analgesia. Anticoagulation was achieved using intraperitoneal heparin (1000 IU/kg/day) administered before surgery and, in recipient animals, during the first 2 postoperative days to prevent anastomotic thrombosis.

In donor rats, a midline laparotomy was performed, and the infrarenal abdominal aorta was carefully dissected free from surrounding tissue; vascular clamps were then placed proximally and distally. A segment measuring approximately 10–15 mm was excised and gently perfused with the assigned cold preservation solution (NTK-Chel, HTK, or normal saline; 4 °C) to flush intraluminal blood. Grafts were subsequently stored in their respective solutions at 4 °C for 2 weeks (Fig. 1). Grafts assigned to the baseline control group were immediately processed for functional and histological analysis without preservation or transplantation. After explantation of the aortic segment, the vascular clamps were released, and the donor animals were euthanized under deep anesthesia by exsanguination.

After 2 weeks of cold storage, preserved grafts were orthotopically transplanted into recipient Wistar rats. Following midline laparotomy and exposure of the infrarenal abdominal aorta, the vessel was cross-clamped and a corresponding segment (~ 10 mm) was excised. The preserved graft was interposed using two end-to-end anastomoses, each constructed with 6–8 interrupted 8–0 nylon sutures. Running sutures were avoided based on prior experience showing increased risk of anastomotic stenosis. Immediately before completion of the distal anastomosis, the distal clamp was briefly released to evacuate intraluminal air and prevent air embolism. The proximal clamp was then removed to restore antegrade blood flow. The abdominal wall was closed in two layers using running 2–0 Vicryl sutures.

Depending on group allocation, recipient rats were re-operated 8, 16, or 26 weeks after implantation under the same anesthesia protocol. Following midline laparotomy, the transplanted aortic segment was carefully dissected and vascular clamps were applied proximally to the cranial anastomosis and distally to the caudal anastomosis. The graft was then completely excised using sharp scissors.

The explanted interposition graft was divided into two equal segments. The proximal segment was immediately placed in Krebs–Henseleit solution (KHS) in an Eppendorf tube for vascular function testing. The distal segment was fixed in 4% buffered paraformaldehyde solution for subsequent histochemical analyses.

After graft explantation, the aortic clamps were released and the animals were euthanized under deep anesthesia by exsanguination.

### Ex vivo vascular reactivity

Aortic segments were mounted as 2-mm ring segments between steel hooks in an organ bath system. One hook was attached to a fixed support, while the second was connected to a movable holder supporting a force transducer (Scientific Instruments, Heidelberg, Germany). Changes in isometric tension were recorded using LabView 6.0 software (National Instruments, Austin, TX, USA). Ring segments were continuously superfused with KHS that was oxygenated (95% O₂, 5% CO₂) and maintained at 37 °C.

After an initial equilibration period (30 min), aortic segments were gradually stretched over 20 min until optimal resting tension of approximately 15 mN/mm was achieved, followed by an additional 20-min equilibration at this tension. All aortic rings were then stimulated with increasing concentrations of KCl (60, 80, 100, and 120 mM) to construct concentration–response curves assessing receptor-independent vasoconstriction.

Vessels that failed to exhibit a contractile response or showed less than 30% of expected maximal contraction were excluded from further analysis. Vessels demonstrating adequate, robust, and reversible contraction were subsequently subjected to receptor-dependent vasoconstriction using stepwise cumulative dosing of phenylephrine, followed by assessment of endothelium-dependent vasodilation using cumulative acetylcholine administration, and finally endothelium-independent vasodilation using sodium nitroprusside.

### Histology and immunohistochemistry

After explantation, aortic segments were fixed in 4% buffered paraformaldehyde solution for 36 h. Segments were dehydrated, embedded in paraffin wax with their longitudinal axis perpendicular to the cutting plane, and sectioned at 5 µm thickness.

Sections were stained with hematoxylin and eosin (H&E) for examination under light microscopy (BH-2, Olympus, Tokyo, Japan). To evaluate intimal hyperplasia, intimal areas were measured by computer-aided planimetry using Analysis 3.2 software (Soft Imaging System, EMSIS, Münster, Germany). Standardized cross-sections from the mid-graft region were analyzed, while anastomotic areas were deliberately excluded to minimize suture-related artifacts and local flow-related distortion. For each graft, the intimal area was determined on the analyzed section, and group data are presented as mean intimal area per group. All analyses were performed in a blinded fashion. 

Immunohistochemical analysis was performed on paraffin-embedded sections to assess morphological changes within the vessel wall. α-smooth muscle actin (α-SMA) staining was used to identify and quantify smooth muscle cells.

Sections were deparaffinized in xylene and rehydrated through graded ethanol (100–30%), followed by washing in distilled water. After rinsing in 0.05 M Tris buffer (pH 7.6), endogenous peroxidase activity was blocked by incubation in TBS⁺ for 30 min in a humidified chamber. Sections were incubated overnight at 4 °C with primary antibody against α-SMA (A2547, Sigma-Aldrich, Merck KGaA, Darmstadt, Germany) at 1:2500 dilution. Following washing, sections were incubated with secondary antibody (rabbit anti-mouse IgG/HRP, P0260, Dako, Glostrup, Denmark) for 30–60 min at room temperature. Immunoreactivity was visualized using 3,3′-diaminobenzidine (DAB) as chromogen, with reaction development monitored visually and stopped by immersion in deionized water. Sections were counterstained with hematoxylin, dehydrated through graded ethanol, cleared in xylene, and mounted using Entellan (Carl Roth GmbH + Co. KG, Karlsruhe, Germany).

α-SMA expression was quantified using Fiji/ImageJ software (National Institutes of Health, Bethesda, MD, USA) as the percentage of α-SMA-positive area within the intima and media of the vessel wall. As described by Navarrete et al. (2020), four equally-sized square regions of interest were placed within the vessel wall at 90° intervals for each vessel. Color deconvolution was performed using the H-DAB algorithm, and α-SMA-positive staining was quantified in the DAB channel using a fixed-intensity threshold applied uniformly across all images. The mean area fraction from the four regions was calculated for each vessel. All analyses were performed blinded to experimental group assignment.

### Statistical analysis

Data were analyzed using GraphPad Prism version 10 (GraphPad Software, Inc., Boston, MA, USA) and are presented as mean ± standard deviation (SD). Data distribution was assessed by visual inspection of box-and-whisker plots and tested for normality using the Shapiro–Wilk test. In cases of small sample sizes, normality was evaluated by visual inspection of residual quantile–quantile (Q-Q) plots derived from analysis of variance (ANOVA) performed on logarithmically transformed data.

For normally distributed data, differences between groups were analyzed using one-way ANOVA, whereas the Kruskal–Wallis test was applied for non-normally distributed data. Post hoc multiple comparisons were performed using Tukey’s test (for parametric data) or Dunn’s test (for non-parametric data), as appropriate. Pairwise comparisons were analyzed using Student’s *t*-test or Mann–Whitney *U* test, and categorical variables were compared using Fisher’s exact test. Survival analysis was performed using the Kaplan–Meier method.

Due to variability and incomplete sigmoidal fitting of individual KCl concentration–response curves, classical dose–response parameters (EC₅₀, Emax) could not be reliably determined. Therefore, overall depolarization-induced contractile capacity was quantified by calculating the area under the concentration–response curve (AUC) for each individual aortic ring using the trapezoidal method with baseline set at zero. Individual AUC values were used for intergroup comparisons. A *p*-value < 0.05 was considered statistically significant.

## Results

### Perioperative data

A total of 73 male Wistar rats of comparable age and body weight were included in the study (Table 3). Statistical analysis revealed no significant differences in donor or recipient body weights among experimental groups. However, animals in the normal saline group exhibited significantly longer anesthesia and anastomosis times compared to the HTK and NTK-Chel groups. This was attributed to inferior graft quality after two weeks of cold storage in normal saline, which rendered the surgical procedure more technically demanding.

**Table 3:** Animal numbers and perioperative characteristics by preservation group and explantation time point. Data are presented as mean with standard deviation (SD). Statistical analysis was performed using ANOVA (ᵃ) and the log-rank test (ᵇ). Abbreviations: *NaCl* normal saline, *HTK* histidine-tryptophan-ketoglutarate, *NTK-Chel N*-acetylhistidine-buffered storage solution enriched with potassium chloride, containing additional amino acids and iron chelators

	Baseline	NaCl	HTK	NTK-Chel	*p*
Animals (*n*)					
8 weeks	3	8	8	10	
16 weeks	3	5	8	5	
26 weeks	3	6	6	6	
Perioperative death		1	1	0	
Total (*n*)	9	20	23	21	
Body weight, mean ± SD, (g)					
Donor	334 ± 15	336 ± 25	332 ± 30	344 ± 25	0.21^a^
Recipient		346 ± 31	348 ± 37	350 ± 20	0.93^a^
Duration (min, mean ± SD)					
Anesthesia		45 ± 6	40 ± 5	41 ± 3	0.0004^a^
Anastomosis		20 ± 3	18 ± 3	18 ± 2	0.04^a^
Survival					
*N* (%)		19 (95)	22 (96)	21 (100)	0.6^b^

All animals in the NTK-Chel group survived the perioperative period. In contrast, one animal each in the HTK and normal-saline groups required euthanasia within 24 h postoperatively due to complete lower-extremity ischemia.

### Vascular contractility

**Fig. 2:**
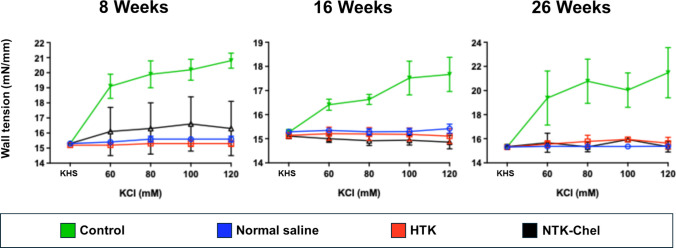
Stepwise potassium chloride (KCl)-induced vasoconstriction assessed by wire myography at 8, 16, and 26 weeks. After equilibration and prestretching in Krebs–Henseleit solution (KHS), vascular rings were exposed to increasing concentrations of KCl (60–120 mM). Wall tension is expressed in millinewtons per millimeter (mN/mm). Data points represent the mean with standard deviation. Experimental groups included baseline control (*n* = 9), NTK-Chel (*n* = 15), histidine-tryptophan-ketoglutarate solution (HTK; *n* = 15), and normal saline (NaCl; *n* = 13). Panels are separated by time point (8, 16, and 26 weeks). HTK, histidine-tryptophan-ketoglutarate; NTK-Chel, *N*-acetylhistidine-buffered storage solution enriched with potassium chloride, containing additional amino acids and iron chelators

Baseline control aortae exhibited robust depolarization-induced contractility, reaching a maximum force of 20.8 ± 0.5 mN/mm at 120 mM KCl (Fig. 2). At 8 weeks post-transplantation, aortic grafts preserved in NTK-Chel demonstrated reduced but detectable contractile responses, with maximal force of 16.8 ± 1.8 mN/mm at 100 mM KCl. In contrast, grafts preserved in normal saline or HTK showed no consistent contractile response to increasing KCl concentrations.

Although NTK-Chel-preserved grafts exhibited partial contractile function, responses did not meet predefined criteria required for subsequent vasomotor analyses. AUC analysis, performed to compare overall depolarization-induced contractile capacity between preservation groups, revealed no significant differences.

At 16 and 26 weeks post-transplantation, none of the transplanted aortic grafts in any preservation group exhibited measurable contractile responses to KCl stimulation. Consequently, further vasomotor analyses were not performed at these time points.

### Histomorphological analysis

#### Intimal hyperplasia

**Fig. 3:**
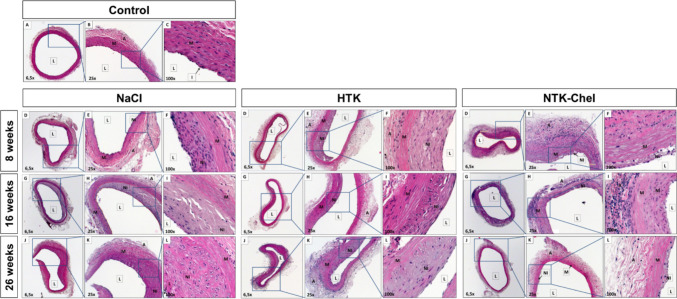
Histological assessment of aortic wall morphology after transplantation and different preservation solutions. Representative hematoxylin and eosin (H&E)-stained cross-sections of aortic segments from the control (native) group and transplanted aortae preserved in NaCl, HTK, or NTK-Chel, analyzed at 8, 16, and 26 weeks after transplantation. For each condition and time point, low-power (6.5 ×), intermediate-power (25 ×), and high-power (100 ×) magnifications are shown. The control aorta demonstrates preserved wall architecture with an intact intima (I), well-organized media (M), and clearly delineated adventitia (A). In transplanted aortae, varying degrees of wall remodeling are observed over time, including changes in medial structure, adventitial thickening, and the presence of neointimal formation. The lumen (L), media (M), adventitia (A), and neointima (NI) are indicated where applicable. Abbreviations: NaCl, normal saline; HTK, histidine-tryptophan-ketoglutarate; NTK-Chel, *N*-acetylhistidine-buffered storage solution enriched with potassium chloride, containing additional amino acids and iron chelators

**Fig. 4:**
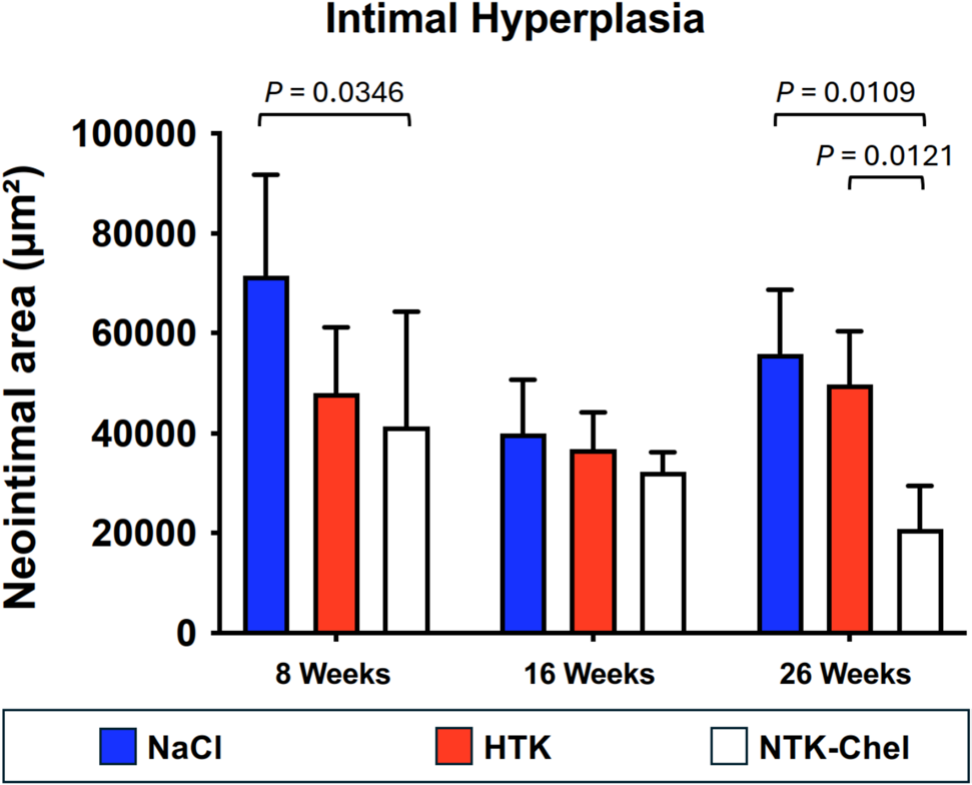
Quantitative analysis of neointimal area after aortic transplantation and different preservation solutions. Grouped bar chart showing the neointimal area (µm^2^) of transplanted aortic segments preserved in NaCl, HTK, or NTK-Chel at 8, 16, and 26 weeks after transplantation. At 8 weeks, the neointimal area was significantly larger in aortae preserved in NaCl compared with NTK-Chel (*p* = 0.0346). At 16 weeks, no significant differences in neointimal area were observed between the groups. At 26 weeks, significant differences were detected between NaCl and NTK-Chel (*p* = 0.0109) as well as between HTK and NTK-Chel (*p* = 0.0121). Data are presented as mean with standard deviation. Group comparisons were performed using one-way ANOVA or the Kruskal–Wallis test, depending on data distribution, with appropriate post hoc testing. Exact *p*-values are shown above the corresponding comparisons. Abbreviations: NaCl, normal saline; HTK, histidine-tryptophan-ketoglutarate; NTK-Chel, N-acetylhistidine-buffered storage solution enriched with potassium chloride, containing additional amino acids and iron chelators

Neointima formation was observed across all preservation groups, with notable differences in severity and temporal progression (Figs. 3 and 4).

In the normal saline group, intimal hyperplasia was evident at 8 weeks, characterized by unstructured and disorganized cellular proliferation. The degree of intimal hyperplasia increased progressively over time, becoming particularly pronounced at 26 weeks. Concomitantly, the medial layer underwent considerable atrophy, comprising solely elastic fibers and extracellular matrix with complete absence of smooth muscle cells.

The HTK group exhibited characteristics comparable to the normal saline group. Distinct intimal hyperplasia was observed at 8 weeks, which showed slight regression at 16 weeks but subsequently increased markedly at 26 weeks. Similarly, the medial layer was notably diminished and devoid of cellular content.

The NTK-Chel group demonstrated the least neointima formation among all groups, particularly at 26 weeks. Moderate intimal hyperplasia was observed at 8 weeks, which gradually decreased over the subsequent time points. Although the medial layer showed notable thinning, the reduction in cellular components was less severe compared to other preservation groups at earlier time points.

#### Smooth muscle cell loss

**Fig. 5:**
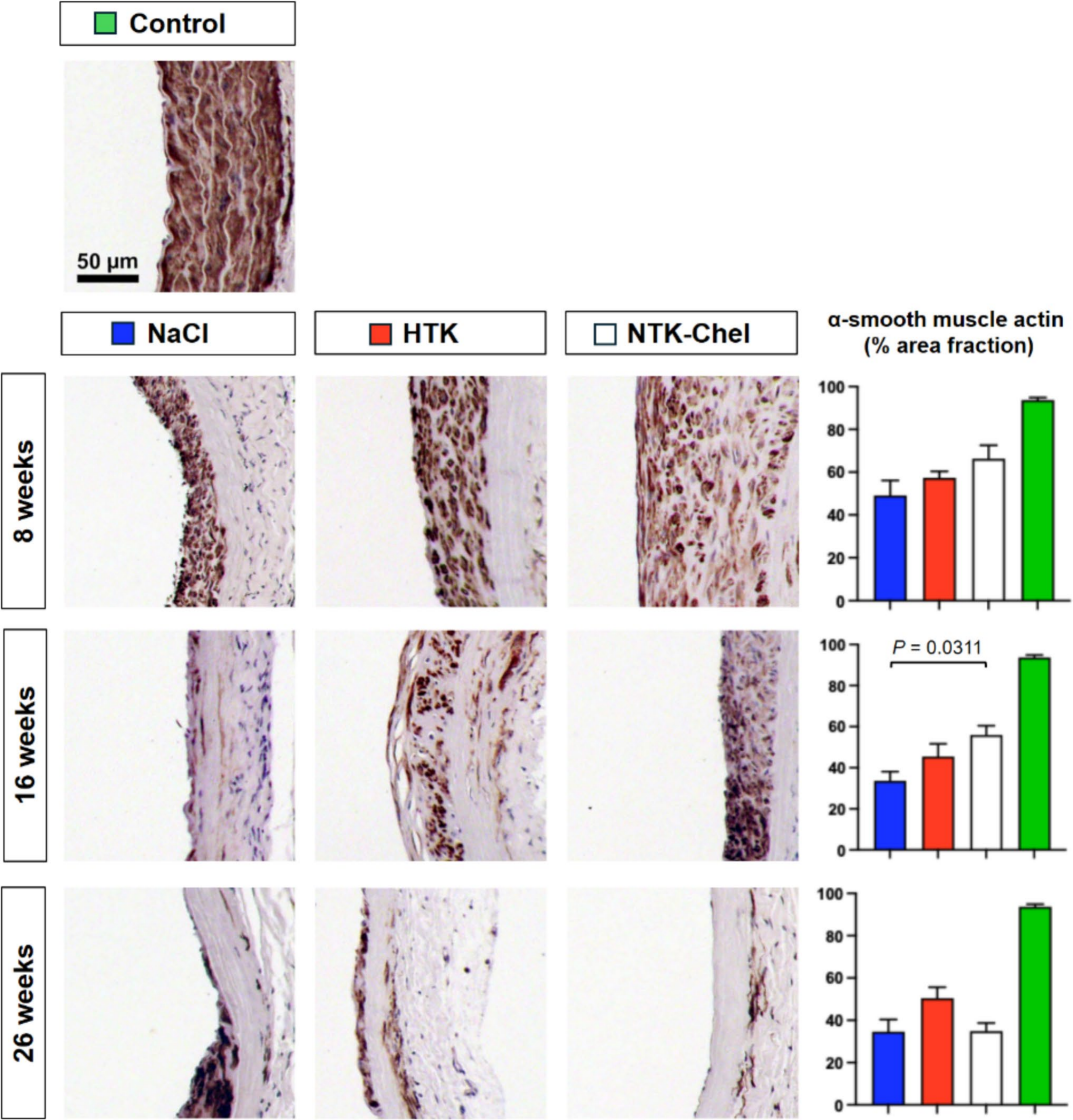
Smooth muscle actin immunohistochemistry and quantitative analysis. Representative images of α-smooth muscle actin (α-SMA) immunohistochemistry of aortic wall sections at 8, 16, and 26 weeks after transplantation. Tissues were preserved using normal saline (NaCl), histidine-tryptophan-ketoglutarate solution (HTK), or NTK-Chel, and compared with baseline (control) aortae. Brown staining indicates α-SMA-positive smooth muscle cells. Quantitative analysis shows the fractional area of α-SMA-positive staining, expressed as a percentage of total tissue area. Data are presented as mean with standard deviation. Group comparisons were performed using one-way ANOVA or the Kruskal–Wallis test, depending on data distribution, with appropriate post hoc testing. Statistical significance between implanted groups is indicated as the exact *p*-value. Abbreviations: NaCl, normal saline; HTK, histidine-tryptophan-ketoglutarate; NTK-Chel, *N*-acetylhistidine-buffered storage solution enriched with potassium chloride, containing additional amino acids and iron chelators

In all experimental groups, the medial layer, normally dominated by smooth muscle cells, showed pronounced reduction in thickness accompanied by marked loss of cellularity (Fig. 5). These changes were heterogeneous both between individual grafts and within the same specimen, with some sections displaying severe medial thinning while the opposing wall remained relatively preserved.

At 8 weeks, α-smooth muscle actin (α-SMA)-positive area was substantially reduced in all transplanted grafts compared with native aorta. This reduction was most pronounced in the normal saline group, showing approximately a 50% decrease relative to baseline controls.

At 16 weeks, medial degeneration progressed in all preservation groups. However, the NTK-Chel group exhibited significantly higher α-SMA-positive area compared with the normal saline group (*p* = 0.031), indicating partial preservation of smooth muscle cells.

At 26 weeks, α-SMA-positive area in the normal saline and HTK groups remained largely unchanged compared with their respective 16-week explants. In contrast, the NTK-Chel group demonstrated marked further reduction in α-SMA staining, reaching levels comparable to the normal saline group. At this time point, no significant differences in α-SMA-positive area were observed between preservation groups.

## Discussion

The principal aim of this study was to evaluate the efficacy of NTK-Chel preservation solution compared to established alternatives (normal saline and HTK) for protecting vascular grafts in a long-term in vivo animal model.

Our main findings demonstrate that NTK-Chel reduced neointima formation also after extended cold storage but that none of the tested solutions fully preserved smooth-muscle-cell function following 2 weeks of cold storage. Vascular contractility was markedly compromised across all experimental groups. However, NTK-Chel showed superior endothelial preservation and notably reduced intimal hyperplasia compared to both normal saline and HTK. This excessive vessel-wall thickening, which frequently leads to luminal narrowing, was least pronounced in the NTK-Chel group at both 8 and 26 weeks post-transplantation.

Since its introduction by Collins et al. in 1969, cold preservation has become standard practice in organ transplantation (Collins et al. 2012). Nevertheless, the cumulative injury from hypoxia, hypothermia, and reperfusion has driven continuous efforts to develop solutions that mitigate these insults, thereby reducing graft failure and extending viable preservation time (Rauen et al. 1999; Salahudeen et al. 2000; Rauen and de Groot 2004). NTK-Chel was developed based on the HTK formulation, incorporating current understanding of hypoxic, hypothermic and reperfusion injury mechanisms. Key modifications include replacing histidine with *N*-acetyl-L-histidine as a buffer and adding the small, cytoprotective amino acids glycine and alanine, and iron chelators, which offer cytoprotection by inhibiting the cold-induced formation of highly reactive oxygen species (Rauen and de Groot 2004; Wille et al. 2008; Zatschler et al. 2009). Inhibiting early injury also reduces the adverse immune response triggered by reactive oxygen species/early reperfusion injury (Perico et al. 2004; Rauen and de Groot 2004; Ali et al. 2018).

Since its introduction, several in vitro and in vivo studies have investigated NTK-Chel’s potential advantages over conventional solutions. However, long-term results remain scarce. The longest in vivo study to date, by Veres et al. (2016), examined aortic allotransplants preserved for 2 h and analyzed 7 days post-transplantation (Veres et al. 2016). Our study therefore provides novel insights into long-term preservation outcomes.

Preservation duration before transplantation is a critical determinant of subsequent graft function and failure risk (Bruinsma et al. 2013). Garbe et al. demonstrated in vitro that NTK-Chel maintains endothelial integrity of human internal mammary arteries superior to conventional solutions for up to 14 days (Garbe et al. 2011). Based on these findings, we selected a 14-day preservation period for the present study.

Despite this rationale, functional vasomotor analysis of transplanted vessels proved largely infeasible due to absent or insufficient contractile responses to high-dose KCl. Notably, in the 8-week group, grafts preserved with NTK-Chel exhibited mild contractile responses, whereas normal saline-preserved and HTK-preserved grafts showed none. Although this response did not meet predefined criteria for subsequent vasoreactivity testing, it may indicate a transient functional advantage of NTK-Chel early after transplantation. This observation is consistent with the notion that preservation solutions primarily exert their protective effects during the early post-transplantation period by mitigating ischemia-reperfusion injury and cold-induced cellular damage. As remodeling processes progressively develop, the initial benefits of superior preservation may become attenuated by mechanisms that operate independently of the original storage conditions.

However, this early advantage was not sustained. At 16 and 26 weeks post-transplantation, no grafts in any preservation group exhibited measurable contractile responses to KCl. The progressive loss of vascular contractility likely reflects multiple contributing factors. Arterial remodeling secondary to allotransplantation shares pathological features with chronic allograft vasculopathy. In long-term rat arterial allograft studies extending to 5 months post-implantation, pronounced loss of medial smooth muscle cells has been described, predominantly mediated by CD8⁺ T-cell-driven immune responses (Mennander et al. 1993; Plissonnier et al. 1995; Légaré et al. 2000). Although our experiments used inbred strains representing a largely syngeneic setting, subtle immunological mechanisms cannot be excluded and may partially account for the observed contractility loss at later time points. This warrants further investigation using autologous graft models.

Additionally, prolonged cold storage itself may contribute to vascular smooth muscle dysfunction. Even under optimized preservation conditions, extended hypothermic storage is associated with cold-induced vascular injury, smooth muscle cell damage, and ischemia–reperfusion stress responses that may irreversibly impair post-implantation vasomotor function.

Histomorphological analysis revealed structural alterations characterized by intimal hyperplasia, particularly pronounced in the normal saline and HTK groups. This phenomenon has been extensively documented and is attributed to reactive oxygen species (ROS), especially during reperfusion (Wei et al. 2023). NTK-Chel-preserved vessels demonstrated significantly reduced long-term endothelial proliferation with better-preserved cellular architecture. This protective effect is attributed to iron chelators, which have been shown to maintain cellular viability and structural integrity (Rauen and de Groot 2004; Veres et al. 2018).

Furthermore, α-SMA staining revealed smooth muscle cell migration into the intima, where they proliferate and contribute to intimal hyperplasia. These smooth muscle cells originate either from the circulation or from adjacent vascular margins (Clowes et al. 1983; Kouchi et al. 1998). Aortic segments stored in normal saline and HTK showed rapid, significant loss of medial smooth muscle cells compared to baseline, leaving only extracellular matrix and elastic lamellae. In contrast, this smooth muscle loss was delayed in the NTK-Chel group, appearing only at 26 weeks. The substantial medial thinning likely reflects two processes: first, smooth muscle cell transformation into myofibroblasts causing medial fibroplasia; and second, smooth muscle cell degeneration resulting in medianecrosis (Batson and Sottiurai 1985; Cao et al. 2022). Importantly, only NTK-Chel-preserved aortic segments showed significantly lower endothelial proliferation compared to normal saline and HTK.

While NTK-Chel preservation may confer early functional advantages, superior endothelial protection and inhibition of intimal proliferation also after extended cold storage, its effects on smooth muscle cells appear only temporarily. It does not prevent smooth muscle cell loss and long-term inflammatory activation or progressive loss of vasomotor function. Whether this less favorable effect of NTK-Chel on smooth muscle cells is due to lower exposure of the smooth muscle cells to the protective components of the solution or whether smooth muscle cells differ from endothelial cells with respect to injurious mechanisms is currently unclear and will have to be addressed in future studies. Additionally, incorporation of very early post-transplantation time points, for instance, 24 h after implantation, in future experiments may help delineate the immediate protective effects of preservation solutions on endothelial integrity and vascular reactivity before remodeling processes become predominant. The absence of a sham group, in which grafts would have been reimplanted immediately without prior cold storage, represents a further limitation, as it precludes differentiation between injury attributable to prolonged hypothermic storage and that resulting from the surgical procedure and subsequent arterial remodeling. The translational relevance of the present model should also be interpreted with caution, as rat aortic grafts differ from human vascular conduits with regard to vessel wall structure, caliber, and hemodynamic exposure. Thus, the present model primarily serves to investigate the biological consequences of prolonged vascular preservation and subsequent implantation under standardized experimental conditions rather than to reproduce a specific clinical scenario.

## Conclusion

The prevalence of peripheral artery disease increases with age, necessitating an increasing number of bypass operations in advanced stages. It is of the utmost importance that a graft is both functionally and morphologically well-preserved to ensure successful long-term revascularization. Previous preservation solutions have been unable to provide adequate protection against cold-induced damage, which is further exacerbated by the dependency on iron. The newly developed NTK-Chel solution addresses this limitation, as evidenced by the results of in vitro studies. The present study demonstrated that NTK-Chel was effective in preventing intimal hyperplasia in comparison to HTK and normal saline solution. This could favour open bypasses in the long term. Notwithstanding the aforementioned advantages, vascular smooth muscle cell function was impaired in all groups following storage, particularly with regard to dilation and contraction.

## Data Availability

All source data for this work, generated in this study, are available upon reasonable request.
